# Piercing and Oral Health: A Study on the Knowledge of Risks and Complications

**DOI:** 10.3390/ijerph17020613

**Published:** 2020-01-18

**Authors:** Francesco Covello, Camilla Salerno, Valentina Giovannini, Denise Corridore, Livia Ottolenghi, Iole Vozza

**Affiliations:** Department of Oral and MaxilloFacial Sciences, “Sapienza” University of Rome, Via Caserta 6, 00161 Rome, Italy; francesco.covello@uniroma1.it (F.C.); camilla.salerno@outlook.it (C.S.); valentina.sciab@yahoo.it (V.G.); livia.ottolenghi@uniroma1.it (L.O.); iole.vozza@uniroma1.it (I.V.)

**Keywords:** oral piercing, oral health, health promotion, dental education

## Abstract

The aim of the present study is to verify the knowledge of risks and complications of oral piercings, and to observe the main complications associated with piercings, using a sample from central Italy of patients wearing intraoral piercings. Through piercing and tattoo studios selected randomly in Rome, Latina and Campobasso, and a tattoo and piercing convention in Latina, a group of 387 individuals with oral piercings were selected and asked to complete an anonymous questionnaire. After filling in questionnaires, 70 individuals of the 387 selected agreed to be visited to allow the observation of the integrity of their teeth and gums (especially close to the oral piercing), oral hygiene conditions, piercing cleaning, bad habits and gingival recession. Among the respondents, 46.8% said they had not been informed about these risks, 48.5% claimed not to clean the piercing, 70.6% stated that they had not been made aware of gingival problems that can arise, 60.4% subjects stated that they were not informed about the complications of piercings concerning teeth, 52.8% had insufficient oral hygiene conditions, 42% showed signs of generalised gingivitis, 20% had 3–4 mm recessions and 22% had tooth fracture(s) due to piercing. From this study, it emerged that oral piercings can represent a risk to oral health and that there is a widespread lack of awareness of the complications and correct methods of maintaining oral piercings. Periodic checks by both dentists and dental hygienists, for patients with oral piercings, could play a decisive role in preventing, intercepting and treating the complications that they can cause.

## 1. Introduction

Piercing is a practice that consists of piercing parts of the body such as ear lobes, nose, eyebrows, navel, nipples and genitals, to insert rings, earrings and piercings. Piercing is a custom that has tribal origins. Since ancient times, it has been used as a form of body decoration, both for purely aesthetic reasons and for ritual reasons, or to affirm one’s belonging to a particular class or ethnic group. 

Today, piercing is very popular among adolescents and young adults as a manifestation of self-expression [1,2]. Several authors have reported the occurrence of undesirable consequences, both minor and important, following skin or mucosal perforation, or due to the constant presence of piercings, both oral and perioral. In 2005, De Moor et al. [3] listed the oral and perioral complications related to the presence of tongue and lip piercings, declaring how fundamental the figure of the dentist was in convincing young people to remove these harmful ornaments [3]. In 2012, studies by Plessas et al. [4] and Ziebolz et al. [5] highlighted dento-periodontal complications caused by piercings in the oral cavity. From these two studies, it emerged how piercings on the tongue led to dental defects, with increased enamel abrasions and dental fractures, and with the presence of gingival recessions on a periodontal level [4,5]. 

Vozza I et al. [6] showed that out of a total of 225 young people who were asked to complete a questionnaire about the oral and systemic complications of oral piercings, 53.7% had not been informed about the risks associated with piercings [6]. The literature also reports how oral piercing can be a possible vector for the transmission of viruses such as HIV, HAV, HBV, HCV, HSV and the Epstein–Barr virus (EBV) [6], as well as causing bacterial pathologies such as Neisseria-induced endocarditis, *Streptococcus viridans* and Ludwig’s Angina [7]. Late complications can lead to bifid tongue, atypical trigeminal neuralgia, soft tongue tissue lesions and hypertrophic keloid lesions [7]. The aim of the present study is to verify the knowledge of risks and complications of oral piercings in a sample from central Italy of patients wearing intraoral piercings, as well as to observe the main complications associated with piercings. 

## 2. Materials and methods

In the present study, through randomly selected piercing and tattoo studios in Rome, Latina and Campobasso, and a tattoo and piercing convention in Latina, a group of 387 individuals with oral piercings were selected and asked to complete an anonymous questionnaire. Information was sought on their knowledge of oral complications related to the insertion and the presence of piercings in the oral cavity. All the individuals that agreed to fill the questionnaire received a consent form and a cover letter that provided information on the objective of the study. Selection of the patients was dependent only on attendance at the studios or the convention previously cited and socioeconomic status, but not on race, religious beliefs or education.

The questionnaire provided was available both in print and online. Names were not recorded on the questionnaire, to ensure anonymity. The study protocol complied with the guidelines of the 1975 Declaration of Helsinki. The ethical authorization was granted by the Research and Ethics Committee of the Sapienza University of Rome.

After filling in the questionnaires, 70 healthy individuals of the 387 selected agreed to be visited. The 70 subjects (18.1%) comprised 53 women and 17 men and were aged between 18 and 40.

The visit, carried out by a single previously trained and calibrated operator at the ASL clinic in Cisterna (Latina), consisted of observing the integrity of teeth and gums, especially close to oral piercings; oral hygiene conditions; piercing cleaning; bad oral habits; and gingival recession. To avoid potential information bias, a clarification was made to the participants that the study would not have any impact on the eventual treatment they sought. Intraoral examinations on all patients were done with the naked eye, using an overhead operating light and a standard mouth mirror. Piercing-related pathology (i.e., tooth fractures, inflammation, infections and chronic lesions) were recorded. The information obtained from the questionnaires was captured in an electronic database, which was verified and validated. Responses to the questions in each category were summarised by calculating the percentages of responses in the respective categories.

## 3. Results

By completing the questionnaire, it was possible to gather a lot of useful data from the 387 selected subjects who participated in the study. There was a higher percentage of individuals aged between 20 and 29 who submitted the questionnaire (64.4%), while 21.1% of individuals were aged between 16 and 19, and only 13.9% were aged between 30 and 39 (Table 1).

Of the 387 selected subjects, 189 (48.8%) reported having a high school diploma, 91 (23.5%) having graduated, 55 (14.2%) having attended a school environment until the age of 14, and 52 (13.5%) having other diplomas/qualifications. Among the other information obtained from the questionnaire, it emerged that 58% of the subjects claimed to be habitual smokers, while in relation to alcohol consumption, 80 subjects (20.6%) declared to be abstainers; 259 (66.9%) claimed to drink alcohol occasionally; and, finally, 48 (12.5%) declared they used alcohol regularly.

It was also interesting to know if any of the subjects had ever undergone orthodontic treatment—228 (59%) subjects replied that they had undergone orthodontic treatment, while 159 (41%) subjects declared they had not.

When patients were asked for information about oral hygiene habits, 219 (56.6%) of the subjects declared that they brushed their teeth at least twice a day; 119 (30.8%) subjects claimed to brush their teeth three times a day; and, finally, 46 (11.9%) subjects admitted to brushing their teeth only once per day. As for the use of dental floss, our survey showed that 307 (79.4%) subjects did not use dental floss, while 80 (20.6%) subjects claimed to use it regularly.

Lastly, the questionnaire focused on the piercings. The results showed that 202 subjects (52.2%), in addition to oral piercings, also had piercings in other areas of the body, while the remaining 185 (47.8%) confirmed that they only had oral piercings. It was in the interest of this study to know where the selected subjects got their piercing done—247 (64%) subjects decided to go to a tattoo and piercing studio; 66 (17%) got their piercing done in a domestic environment; 38 (9.8%) opted for a jewellery store; and, finally, 21 (5.4%) performed self-piercing.

Very important information collected from the questionnaire concerned the reason why these young people decided to get the piercing. Expressing their personality was reported by 117 (30.2%), 170 for aesthetic reasons (43.9%), 51 for erotic reasons (13.2%), 45 for fashion (11.6%) and four because of the influence of friends (1%). Furthermore, 53.2% said they were informed about the complications of piercings on their general health, while 46.8% said they had not been informed about them (Table 1).

When asked about cleaning of the piercing, 188 subjects (48.5%) claimed not to clean the piercing, while 199 (51.5%) stated that they do so regularly (Table 2). With regard to the methods used for cleaning the piercing; 155 subjects (40%) used a brushing technique and an antimicrobial solution; 124 (32%) declared only using the antimicrobial solution; and, finally, 108 (28%) confirmed only using the brushing technique. 

Regarding the knowledge of possible gingival complications due to piercings, 273 subjects (70.6%) stated that they had not been made aware of gingival problems that can arise, while 114 subjects (29.4%) confirmed that they had been made aware of this. With regard to the dental field, 234 (60.4%) subjects stated that they were not informed about the complications of piercings concerning teeth, while 153 (39.6%) subjects claimed to have been informed of these complications (Table 2). 

Alongside completing the questionnaire, 70 subjects aged between 18 and 40 with one or more oral piercings were visited. During the visits, we wanted to consider the areas where the piercing was located and their oral hygiene conditions. The age group with the highest number of subjects visited was those aged 20–29, with a percentage of 80%; followed by the 30–39 age group, with a percentage of 10%; then the 16–19 age group, with a percentage of 7.5%; and, finally, the 40–49 age group with a percentage of 2.5%. 

Regarding the location of oral piercings, it should be pointed out that some subjects had more than one piercing present in the oral cavity. Our visits showed that 42 patients had piercings on their tongue, 17 on their lip (lower or upper) and 20 on their frenulum. We also wanted to identify whether the subjects visited had bad habits. All the sample patients taken into consideration had admitted to having one or more of the following bad oral habits: smoking, nail biting, playing with oral piercing using their tongue or making it bang against their teeth and lip biting. More precisely, 49 subjects reported regular smoking (70%), 40 (57.1%) confirmed constantly playing with the oral piercing, 24 subjects (34.3%) declared biting their nails and 25 admitted biting their lips (35.7%). 

**Figure 1:**
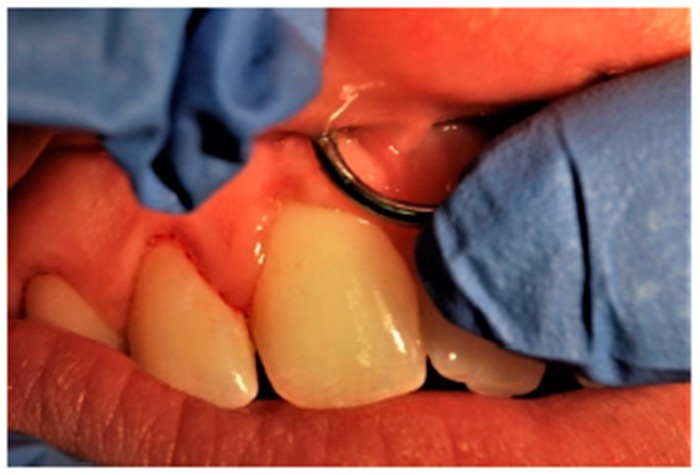
Clinical gingival recession.

**Figure 2:**
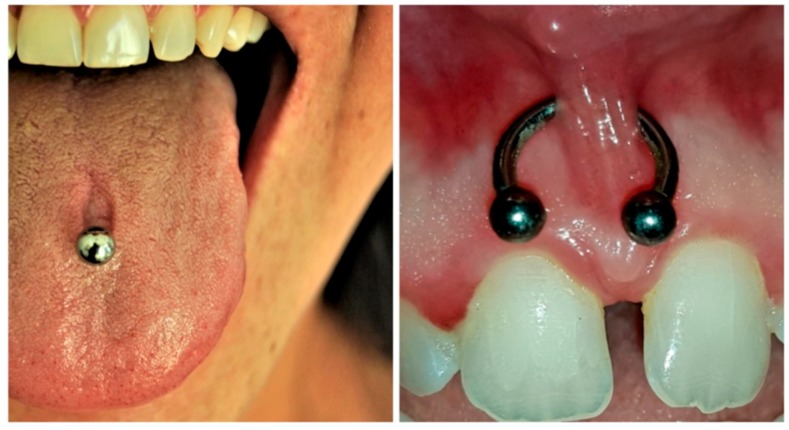
Tongue lesion and thickening of frenula.

**Figure 3:**
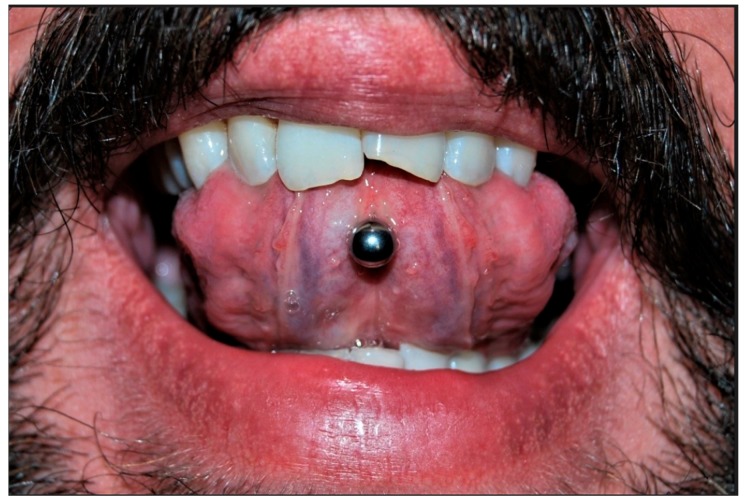
Fractured upper central incisor.

When the oral hygiene condition of each patient were examined, 15 subjects (21.4%) showed good oral hygiene condition, 18 (25.7%) barely sufficient, and 37 (52.8%) had insufficient oral hygiene condition. Precisely for this reason, most of the subjects visited had gingivitis, either localised or generalised. In 31 subjects (44%), the presence of localised gingivitis was observed, almost always close to their piercing, while 29 patients (42%) showed signs of generalised gingivitis. Only 10 patients did not present the classic signs of gingival inflammation (14%). Table 2 Another complication evaluated during visits was the presence of gingival recession. Almost all subjects examined showed at least one gingival recession. Recessions of about 1–2 mm were found in 46 subjects (65%); 3–4 mm recessions in 14 subjects (20%); and, finally, 10 subjects (15%) showed gingival recessions of 5–6 mm (Table 3). A part-gingival recession was found (Figure 1), as well as other oral complications such as a tongue lesion and thickening of the frenula (Figure 2). Among the subjects visited, 22% showed a fractured tooth (Figure 3). The fractures, as reported by patients, were caused by continuous playing with the piercing, which led to dental elements being repeatedly hit. The teeth most compromised by the presence of piercings were the molars, but incisors and premolars were also seriously damaged.

## 4. Discussion

The results of our study suggest that, despite the increasing number of young people becoming interested in body art, there is still not enough awareness of the complications that piercing involves. As demonstrated in a study by Vozza I et al. [8] in 2015, these problems are still not known well by the piercers themselves, whose hours of professional training differ from region to region. No correlation between age or school education and oral complications occurred in our study. From the data found through the questionnaire, it emerged that many piercers do not always inform their clients about what may be the general complications related to piercing. Specifically, many of them are unable to tell customers about the complications that piercings can cause to teeth, such as fractures, and gums, such as gingival recession or frenula thickening, as well as how to clean them, which is of vital importance in order to prevent gingivitis or periodontitis [9]. Consequently, the dentist and the dental hygienist have to deal with the task of informing their patients with piercings of the aforementioned information. The importance of the dental team was evaluated by Maspero C. et al. [10] in 2014. Our study confirmed information found in literature [11,12,13,14] describing how oral piercings are the cause of a series of complications, such as dental abrasions, dental fractures, gingival recessions and loss of attachment of periodontal tissues, but also problems related to temporomandibular joint, as stated by Mejersjö in 2016 [15]. It is therefore necessary to establish education programs in schools between dentistry students and dentists themselves, in order to prevent the aforementioned complications reported, respectively, by Silk H et al. [16], Junco P et al. [17] and McGeary SP et al. [18].

## 5. Conclusions

From this study, it emerged that oral piercings can represent a risk to oral health, as found by Maitland I et al. [19]. There is a widespread lack of awareness regarding the complications and correct methods of maintaining oral piercings, as confirmed in the literature, among dental students [20]. Compared to our previous study on adolescents and young adults [6], lack of knowledge was surveyed among older individuals, comparing results of questionnaires to patients wearing oral piercings and directly visited. In light of our research, it is clear that projects regarding oral health and prevention, together with periodic checks by both dentists and dental hygienists, for patients with oral piercings can play a decisive role in preventing, intercepting and treating the complications that they can cause. Further studies will be necessary in order to establish a statistical correlation between oral piercing and oral complications that consider age, sex and education.

## Figures and Tables

**Table 1:** Characteristics of the sample, resulting from the administration of 387 questionnaires.

	Percentage
Age	
16–19	21.10%
20–29	64.40%
30–39	13.90%
40–49	0.60%
Smoker and alcohol consumption	
Habitual smoker	58.0%
Abstainer	20.6%
Drink alcohol occasionally	66.9%
Use alcohol regularly	12.5%
Undergone orthodontic treatment	
Yes	59.0%
No	41.0%
Oral hygiene habits related to toothbrushing	
Once a day	11.90%
Twice a day	56.60%
Three times a day	30.80%
More than three times a day	0.70%
Places where piercings were performed	
Tattoo and piercing studios	64%
Cosmetic doctor	3.80%
Self-insertion	5.40%
Home environment	17.0%
Jewellery store	9.80%
Reason for getting piercing	
Express their personality	30.2%
Aesthetic reason	43.9%
Erotic reason	13.2%
Fashion	11.6%
Conditioned by friends	1.0%

**Table 2:** Level of knowledge of piercing management, resulting from the administration of 387 questionnaires.

	Percentage
Subjects informed about general health related to piercing	
Uninformed subjects	46.8%
Informed subjects	53.2%
Cleaning habit related to piercing	
Not cleaning	48.5%
Cleaning regularly	51.5%
Methods used for cleaning	
Brushing and antimicrobial solution	40.0%
Antimicrobial solution	32.0%
Brushing technique	28.0%
Subjects informed about gingival complications related to piercing	
Uninformed subjects	70.6%
Informed subjects	29.4%
Subjects informed about dental complications related to piercing	
Uninformed subjects	60.4%
Informed subjects	39.6%

**Table 3:** Periodontal conditions of the sample collected after clinical examination of 70 subjects.

	Percentage
Age	
16–19	7.5%
20–29	80.0%
30–39	10.0%
40–49	2.5%
Piercing placement	
Tongue	53.0%
Lip	22.0%
Frenula	20.0%
Bad habits	
Regular smoking	70.0%
Playing with piercing	57.1%
Biting nails	34.3%
Biting lips	35.7%
Oral hygiene condition	
Good	21.4%
Sufficient	25.7%
Insufficient	52.8%
Gingival condition	
Localised gingivitis	44.0%
Generalised gingivitis	42.0%
No gingival inflammation	14.0%
Gingival recession	
1–2 mm	65.0%
3–4 mm	20.0%
5–6 mm	15.0%
